# Relationship of bruxism with oral health-related quality of life and facial muscle pain in dentate individuals

**DOI:** 10.4317/jced.59255

**Published:** 2022-05-01

**Authors:** Karina-Helga-Leal Turcio, Clóvis-Lamartine-de Moraes-Melo Neto, Beatriz-Ommati Pirovani, Daniela-Micheline dos Santos, Aimée-Maria Guiotti, André-Pinheiro-de Magalhães Bertoz, Daniela-Atili Brandini

**Affiliations:** 1 DDS, MS, PhD. Department of Dental Materials and Prosthodontics, Araçatuba Dental School, São Paulo State University (UNESP), São Paulo, Brasil; 2 DDS, MS. Department of Surgery and Integrated Clinic - Divison of Periodontics, Araçatuba Dental School, São Paulo State University (UNESP), São Paulo, Brasil; 3 DDS, MS, PhD. Oral Oncology Center, São Paulo State University (UNESP), Araçatuba Dental School, São Paulo State University (UNESP), São Paulo, Brasil; 4 DDS, MS, PhD. Department of Pediatric and Social Dentistry, São Paulo State University (UNESP), School of Dentistry, Araçatuba, São Paulo, Brasil; 5 DDS, MS, PhD. Department of Diagnosis and Surgery, Araçatuba Dental School, São Paulo State University (UNESP), São Paulo, Brasil

## Abstract

**Background:**

To determine whether there is a correlation of bruxism (sleep, daytime, or both) with oral health-related quality of life and facial pain of muscular origin in dentate individuals.

**Material and Methods:**

Seventy-four dentate patients (complete dentition) were included in this study. These individuals had pain in the facial muscles due to temporomandibular disorder (TMD). Smokers; and those with obstructive sleep apnea, TMD of joint origin associated or not with pain, malocclusion, and cancer; and users of illicit drugs, psychiatric medications, and alcohol were excluded. Obstructive sleep apnea, bruxism (of sleep and/or daytime), facial muscle pain, and oral health-related quality of life were assessed by the following questionnaires: Berlin Questionnaire, Pintado *et al*. questionnaire, VAS (Visual Analog Scale) facial muscle pain questionnaire, and Oral Health Impact Profile – 14. Four groups were created: 1) no bruxism; 2) sleep bruxism; 3) daytime bruxism; and 4) sleep and daytime bruxism. Spearman’s correlation test was applied to verify if there was a correlation between the collected data. *P* values less than 0.05 were considered statistically significant.

**Results:**

There was a positive correlation of daytime bruxism with mean pain in the last 3 months (*P*<0.05) and the worst pain experienced in the last 3 months (*P*<0.05).

**Conclusions:**

Bruxism (sleep, daytime, or both) showed a positive correlation with lower oral health-related quality of life (*P*<0.05).

** Key words:**Bruxism, facial pain, temporomandibular joint disorders, surveys and questionnaires, health-related quality of life.

## Introduction

The term “temporomandibular disorders” (TMD) is often used to denote musculoskeletal disorders in the jaw muscles and/or the temporomandibular joints (1). Bruxism can cause facial pain of muscle origin (2).

Bruxism may be classified as daytime or sleep bruxism (3,4). According to the ninth edition of the prosthodontic glossary, bruxism may be defined as “1- parafunctional grinding of teeth; 2- an oral habit consisting of involuntary rhythmic or spasmodic nonfunctional gnashing, grinding, or clenching of teeth, in other than chewing movements of the mandible, which may lead to occlusal trauma” (5).

The etiology of bruxism is not completely clear (3). It is suggested that bruxism can be caused by four groups of factors: Group 1 – Biologic factors (which include neurotransmitters, e.g., dopamine) and genetic factors; Group 2 – Factors of exogenous origin, which include nicotine, caffeine, alcohol, illicit drugs, and some medication (e.g. fluoxetine); Group 3 – Psychologic factors, which include sensitivity to stress, individual character traits, and anxiousness, among others (6), and Group 4 - Sleep disorders (e.g., obstructive sleep apnea) (3). Thus, there is no single factor responsible for bruxism.

Jaw muscle pain is the most common type of pain in TMD patients (1). This pain is often chronic, and includes the characteristics of pain at rest and pain that exacerbates during jaw functions such as biting, chewing, and yawning (1). Thus, this type of TMD must be studied.

The purpose of this study is to determine whether there is a correlation of bruxism (sleep, daytime, or both) with oral health-related quality of life and facial pain of muscular origin in dentate individuals.

## Material and Methods

-Ethics Committee

This cross-sectional study was approved by the ethics committee (number: 53193216.5.0000.5420) of the Araçatuba Dental School (São Paulo State University), and it was carried out between 2016 and 2017 in accordance with the Declaration of Helsinki (7). All participants were informed about the study and signed a free and informed consent form.

-Patient selection 

One hundred and thirty patients from the nucleus for the diagnosis and treatment of TMDs (Araçatuba Dental School - São Paulo State University) were invited to participate in this research.

All selected patients were evaluated by the same professional, who had experience with TMDs. After the patients were evaluated based on the inclusion and exclusion criteria, 74 patients with TMD of muscle origin were included in this study.

-Inclusion criteria

•Men and women over 18 years of age.

•Complete dentition.

•TMD of muscular origin, verified by the RDC/TMD (Research diagnostic criteria for temporomandibular disorders) Axis I questionnaire (8).

•Individuals must not be using a muscle relaxant medication chronically in the last 3 months.

-Exclusion criteria

•Individuals with a high probability of possessing obstructive sleep apnea (OSA), as OSA can cause sleep bruxism (9-11). In addition, OSA can cause sleep disturbances (9), and poor sleep quality may be related to an increase in pain sensitivity (12).

•Individuals who used a bite plate at night due to sleep bruxism.

•TMD of joint origin associated or not with pain.

•Presence of malocclusion. Some types of malocclusion can lead to bruxism and TMD. (3,13).

•Difference between centric relation and maximum intercuspation greater than 5 mm (13).

•Use of psychiatric medication, as could induce bruxism (4,6,14) and alter sleep patterns (9,15).

•Abuse of alcohol consumption (6).

•Use of illicit drugs (4,6).

•Smokers (6).

•Cancer (16).

•Those that were not willing to participate in the study.

After the inclusion of patients in this study, questionnaires about the presence of bruxism, facial pain, and oral health-related quality of life were applied.

-Berlin Questionnaire

The Berlin Questionnaire used in the present study was validated for the Portuguese language (Brazil) (17). This questionnaire is a screening tool used to differentiate individuals with a high or low chance of having OSA. This questionnaire consists of 10 items, divided into three categories (1. snoring and witnessed apnea, 2. daytime sleepiness, and 3. arterial hypertension/obesity). According to Andrechuk *et al*.: Category 1 ranges from 0 to 6 points and is considered positive when the score is 2 points or more; Category 2 ranges from 0 to 3 points and is considered positive when the score is 2 points or more; and Category 3 will be positive if the participant reports that he/she has high blood pressure or a body mass index (BMI) >30 kg/m2 (17). The final score indicates a high risk of OSA when two or more categories are positive. A low risk of OSA is indicated when all of the categories had no positive score or when there was a positive score in only one category.

-Pintado *et al*. questionnaire

The identification of bruxism was based on the Pintado *et al*. questionnaire (18), involving the following questions: 1. Has anyone heard you grinding your teeth at night?; 2. Is your jaw ever fatigued or sore on awakening in the morning?; 3. Are your teeth or gums ever sore on awakening in the morning?; 4. Do you ever experience temporal headaches on awakening in the morning?; 5. Are you ever aware of grinding your teeth during the day?; and 6. Are you ever aware of clenching your teeth during the day? Each participant could have responded “yes” or “no” for each question, and one positive response (“yes”) already classified the individual as having sleep bruxism, daytime bruxism, or both types of bruxism (of sleep and daytime).

-VAS (Visual Analog Scale) facial muscle pain questionnaire 

The presence of muscle pain was assessed using a Visual Analog Scale (VAS) according to the RDC/TMD Axis I questionnaire (8). Patients were instructed to answer each item by marking a vertical line on a 10-centimeters horizontal scale.

VAS facial muscle pain questionnaire.

1. How would you rate your facial pain on a 0 to 10 scale at the present time, that is right now, where 0 is “no pain” and 10 is “pain as bad as could be”?

2. In the last 3 months, how intense was your worst pain rated on a 0 to 10 scale where 0 is “no pain” and 10 is “pain as bad as could be”? 

3. In the last 3 months, on the average, how intense was your pain rated on a 0 to 10 scale where 0 is “no pain” and 10 is “pain as bad as could be”? [That is, your usual pain at times you were experiencing pain].

-Oral Health Impact Profile – 14

Oral health-related quality of life was assessed using the OHIP-14 (Oral Health Impact Profile – 14) questionnaire (19). The OHIP-14 questionnaire is composed of 14 questions that assess the following situations: functional limitation, physical pain, psychological discomfort, physical disability, psychological disability, social disability, and handicap. For each question, the patient must choose one of the following answers: 0 = never, l = hardly ever, 2 = occasionally, 3 = fairly often, or 4 = very often. Subsequently, for each question, the patient’s answer score is multiplied by the weight of the question. It is noteworthy that each question has a specific weight for itself. Also, for this questionnaire, the word “dentures” was replaced by “face muscles”.

For this questionnaire, the maximum value of the sum of scores is 28 (total score) and the minimum value of the sum of the scores is 0 (total score). Thus, the higher the total score, the lower the individual’s oral health-related quality of life.

-Statistical analysis

After data collection, four groups were created: 1) no bruxism; 2) sleep bruxism; 3) daytime bruxism; and 4) sleep and daytime bruxism. Then, the data were submitted to statistical analysis using the IBM SPSS 20.0 (Statistical Package for the Social Sciences, IBM, USA). Spearman’s correlation test was applied to verify if there was a correlation between the collected data. *P* values less than 0.05 were considered statistically significant.

## Results

Among the 74 selected patients, 60 (81.08%) were female and 14 (18.92%) were male. The age of individuals ranged from 18 to 78 years.

Table 1 shows the number of participants in each group. Table 2 shows the mean results of self-reported pain and oral health-related quality of life for the groups.

**Table 1:**
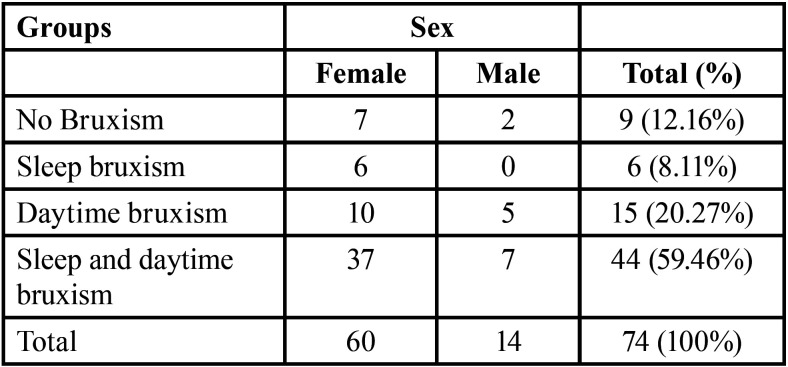
Number of individuals in each group.

**Table 2:**
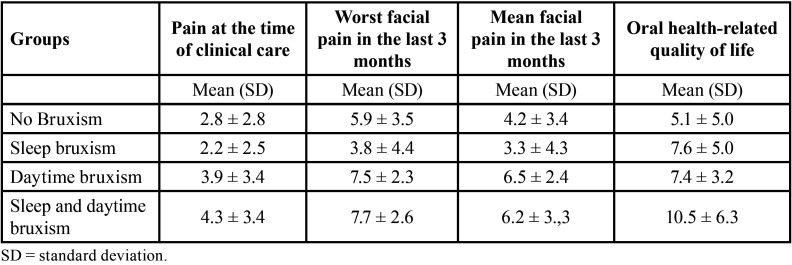
Mean results (standard deviation) of self-reported pain and oral health-related quality of life.

Table 2 shows the results obtained with the OHIP-14 and VAS facial muscle pain questionnaires.

There was a positive correlation of daytime bruxism with the worst pain experienced in the last three months (*P*<0.05) and the mean facial pain in the last three months (*P*<0.05). In addition, there was a positive correlation between the presence of bruxism (daytime bruxism, sleep bruxism, and both types of bruxism) and high scores for the OHIP-14 questionnaire (*P*<0.05) (Table 3).

**Table 3:**
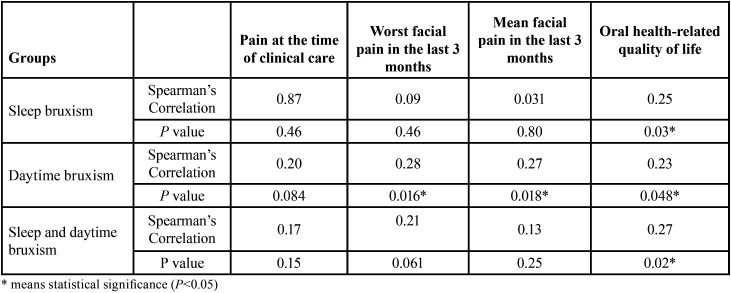
Spearman’s correlation of the type of bruxism with facial pain and oral health-related quality of life.

## Discussion

Full-night polysomnography is the gold standard for diagnosing sleep bruxism (20). Despite this, this test was not used in the present study due to the high costs involved.

In this study, based on bruxism-related groups (daytime bruxism, sleep bruxism, and both types of bruxism), the majority of participants were female (Table 1). Women are more affected by stress factors than men, since they consider these stresses more threatening (21). This situation may explain the higher prevalence of women in these groups (Table 1), because: 1 - daytime bruxism could be considered a response to stress and anxiety (22,23); and 2 - sleep bruxism may also be associated with psychological factors such as stress (23-26).

There was a positive correlation of daytime bruxism with the worst pain experienced in the last three months (*P*<0.05) and the mean facial pain in the last three months (*P*<0.05) (Table 3). Therefore, the more daytime bruxism is performed, the greater the facial pain and vice versa. In addition, there was a positive correlation between the presence of bruxism (daytime bruxism, sleep bruxism, and both types of bruxism) and high scores for the OHIP-14 questionnaire (*P*<0.05) ( Table 2, Table 3). Therefore, the more bruxism (sleep bruxism, daytime bruxism, or sleep and daytime bruxism) is performed, the lower the oral health-related quality of life and vice versa. Despite this, these correlations are considered weak (27).

Sleep bruxism can be characterized by tonic (teeth clenching) and/or phasic (teeth grinding) activity, and daytime bruxism is predominantly characterized by tonic activity (28). The results of this study did not show a correlation between facial pain and sleep bruxism (*P*>0.05) ( Table 3). This happened because in the “sleep bruxism group” possibly the phasic activity (teeth grinding - brief, repeated contractions of the masticatory musculature) predominated, as opposed to the “daytime bruxism group” in which the tonic activity (teeth clenching - sustained for more than two seconds) was possibly predominant (28). Thus, due to the fact that sustained contraction causes greater and faster muscle fatigue than brief contractions, this may be a factor that explains this result ( Table 3). It is noteworthy that there was also no correlation between facial pain and both types of bruxism. Possibly, in the group with both types of bruxism, sleep bruxism was predominant, as well as phasic activity.

## Conclusions

There was a positive correlation of daytime bruxism with mean pain in the last 3 months and the worst pain experienced in the last 3 months. Bruxism (sleep, daytime, or both) showed a positive correlation with lower oral health-related quality of life.
